# Effect of Leukoreduction by Pre-Storage Filtration on Coagulation Activity of Canine Plasma Collected for Transfusion

**DOI:** 10.3390/vetsci8080157

**Published:** 2021-08-04

**Authors:** Eva Spada, Roberta Perego, Luciana Baggiani, Daniela Proverbio

**Affiliations:** Veterinary Transfusion Research Laboratory (REVLab), Department of Veterinary Medicine (DIMEVET), University of Milan, 26900 Lodi, Italy; luciana.baggiani@unimi.it (L.B.); daniela.proverbio@unimi.it (D.P.)

**Keywords:** canine, transfusion, plasma, leukoreduction, coagulation

## Abstract

Leukoreduction of blood products is a technique used to prevent leukocyte-induced transfusion reactions and is extensively used in human, but rarely in veterinary patients. The concentration of some coagulation proteins can be affected by the processing steps used for the preparation of leuko-reduced plasma units. In this study, we assessed the effect of leukoreduction on coagulation activity of canine plasma collected for transfusion. Ten plasma units, five obtained from non-leuko-reduced (non-LR) whole blood (WB) units and five from leuko-reduced (LR) WB units were evaluated. Prothrombin time (PT), activated partial thromboplastin time (aPTT), coagulation factor activities of factors (F) V, VIII, X, XI, and von Willebrand (vWF), fibrinogen and D-dimers content were assessed at collection (baseline value, D0) and after 7 days of frozen storage at −18 °C (D7). Compared to non-LR plasma units, LR units showed a statistically significant prolonged aPTT and reduced FXI activity. Filtration had no significant effect on the other factors and parameters evaluated. Filtration-dependent changes appear to have no impact on the therapeutic quality of plasma obtained from leuko-reduced whole blood, other than for FXI activity. Further studies on a larger sample size comparing the same unit before and after leukoreduction are needed to confirm these findings.

## 1. Introduction

Transfusion medicine has grown in recent decades and many canine patients today receive blood or blood components. Plasma components, such as fresh frozen plasma (FFP), are used mainly to treat coagulopathies [1,2,3]. The plasma contains therapeutic levels of functional coagulation factors, transfusion of which is an essential component of treatment for many congenital and acquired coagulopathies [4,5].

Blood or plasma transfusions have the potential to cause severe and potentially life-threatening reactions in recipients. Some of these reactions are due to the presence and activities of leukocytes. For this reason, in human medicine in almost all countries, blood components undergo leukoreduction to prevent a number of leukocyte-induced transfusion reactions, such as febrile transfusion reactions [5].

While leukoreduction of blood products is extensively used in human medicine, as demonstrated by the numerous papers published on leukoreduction [6,7,8,9,10,11,12,13,14,15,16,17], it is still rarely used or reported in veterinary medicine, where leukoreduction is usually achieved by filtration of whole blood (WB) before blood components are separated [18,19,20,21,22,23,24]. Based on evidence in human studies that leukoreduction decreases the rate of febrile non-hemolytic transfusion reactions, a recent Transfusion Reaction Small Animal Consensus Statement suggested that leukoreduction should also be considered in veterinary medicine [25], even though there is currently insufficient evidence to show that the use of leukoreduction prevents or reduces any type of transfusion reaction in veterinary medicine. To date, there has been only one study in normal healthy dogs that clearly showed that the inflammatory response to transfusion is eliminated by leukoreduction [18].

The concentration of some coagulation proteins can be affected by the leukoreduction. Many published human studies have investigated the effect of leukoreduction on residual clotting factors of either whole blood or plasma, but have shown contradictory results. Some studies report a significant effect on level and activity of some coagulation factors [6,7,8,9], whilst others reported no significant effect [10,11,12,13,14,15]. One study even reported that pre-storage leukocyte depletion improved the coagulation factor content of plasma in stored whole blood [17]. In veterinary medicine, one study highlighted a variation among residual activities of coagulation factors in LR and non-LR canine FFP, with variations and differences that were considered unlikely to impact the efficacy of LR FFP transfused for coagulation factor replacement [26].

Therefore, when evaluating a leukoreduction system, it is essential to assess not only the ability of the system to remove leucocytes, but also to assess whether treatment will change the quality of the final product. Given the therapeutic value of plasma, it is important that filtration of blood or plasma does not remove a significant percentage of coagulation factors or inhibitors of coagulation. Therefore, it is equally important to investigate activation of coagulation and inflammatory pathways that may increase the incidence of side-effects such as febrile reactions or anaphylaxis. During filtration, blood comes into contact with the artificial surface of the filters, some of which are negatively charged, which could result in activation of the coagulation system [16] or removal of complement system inhibitors [9].

Most published veterinary reports have studied the efficacy of blood filters for reducing the leukocyte count in final blood components [22,24,26], and their effect on RBC viability and on reduction of red cell storage lesions [19,20,21,22,23,24,27,28,29,30]. However, little is known about the effect of leukoreduction on the hemostatic activity of plasma obtained for transfusion purposes and, to the author’s knowledge, only two veterinary studies have evaluated this using human and canine blood collection systems [24,26]. These studies found no significant change in FFP obtained from LR WB units other than a prolonged activated partial thromboplastin time (aPTT) [24]. The objective of this study was to evaluate the concentration of canine hemostatic proteins, including adhesive proteins, selected clotting factor activities and anticoagulant factors in plasma units obtained using a canine collection bag system and WB pre-storage filtration. The hypothesis for this study was that the LR plasma would have lower concentrations of some coagulation factors, compared with non-LR plasma, similar to the effects reported by some human studies [6,8,9].

## 2. Materials and Methods

### 2.1. Blood Donors, Sample Collection and Processing

In this prospective study, donor dogs were recruited from the pre-established blood donation program, including client-owned dogs from the Veterinary Transfusion Research Laboratory (REVLab) of the University of Milan. Blood units were collected with the consent of each donor dog owner.

WB units were collected from ten privately owned, Dog Erythrocyte Antigen (DEA) 1-positive (7/10 dogs also tested DEA4-positive and DEA7-negative), Golden Retrievers. The donors were 9 intact males and 1 intact female. The mean age of the dogs was 4 years (±1.9 years, range 2–8 years). Each dog weighed >25 kg, was free from systemic and infectious diseases, was deemed healthy based on routine pre-donation screening (history, physical exam and results of a CBC, biochemical analysis and vector-borne diseases tests) and was not taking any medication known to interfere with coagulation testing.

Whole blood (WB) units (200 mL), obtained in accordance with the standard operating procedures of the REVLab Volunteer Blood Donor Program, were collected using specific canine bag collection systems (AB bags, TEC 710 and TEC 709 Futurlab, Limena, Italy) containing CPDA-1 (citrate, phosphate, dextrose and adenine) as an anticoagulant in a ratio of 1:7 with collected blood. Briefly, the blood collection system was placed on the gram scale and the tubing was temporarily clamped to prevent air from entering the bag when the needle cap seal was broken. The non-sedated donor was positioned in either lateral or sternal recumbency. Jugular or cephalic vein access was clipped, and the skin was aseptically prepared to prevent bacterial contamination. Venipuncture was performed using the 16-gauge needle attached to the connecting tube of the collection system. The clamp on the bag was released from the tubing and blood flowed into the blood collection system by gravity assistance, until approximately 200 mL of blood had been collected. Then, the blood line was re-clamped close to the needle and the needle was removed from the donor. Pressure was applied to the venipuncture site, first digitally, and then with a pressure bandage, until hemostasis was achieved. Meanwhile, a line stripper was used to move the blood in the tubing into the collection bag, which was then gently rocked to mix the blood with the anticoagulant.

Only plasma units were used in this study, and the remaining components (i.e., packed red blood cells (pRBC)) were used for transfusion purposes.

All ten WB units were collected on the same day and were stored refrigerated at 2–6 °C for approximately 2 h until all donations were complete. Five randomly chosen WB units were not leuko-reduced (non-NR) and five were leuko-reduced (LR) prior to separation of WB units into pRBCs and plasma units. For leukoreduction, WB units were hung with the tubing extended to allow filtration to occur via gravity flow and filtered at room temperature (20 °C) using an integrated inline whole-blood leukocyte reduction filter for single use made from non-woven polyester fibers (Leucolite^TM^, GLT Medical Co., Ltd., Zhangjiagang, China) in the specific canine collection system (TEC 710 Futurlab, Limena, PD, Italy). This process took approximately 15 min. The leukoreduction filter used in this study had previously been evaluated [24] and yielded residual leucocyte counts below 1 × 10^6^ WBC per unit, and an almost complete platelet depletion, as required by the European human guideline for quality of FFP and pRBC [31]. This also fulfilled the US Food and Drug Administration (FDA) requirements of a residual number of <5.0 × 10^6^ leukocytes per unit [32].

**Figure 1:**
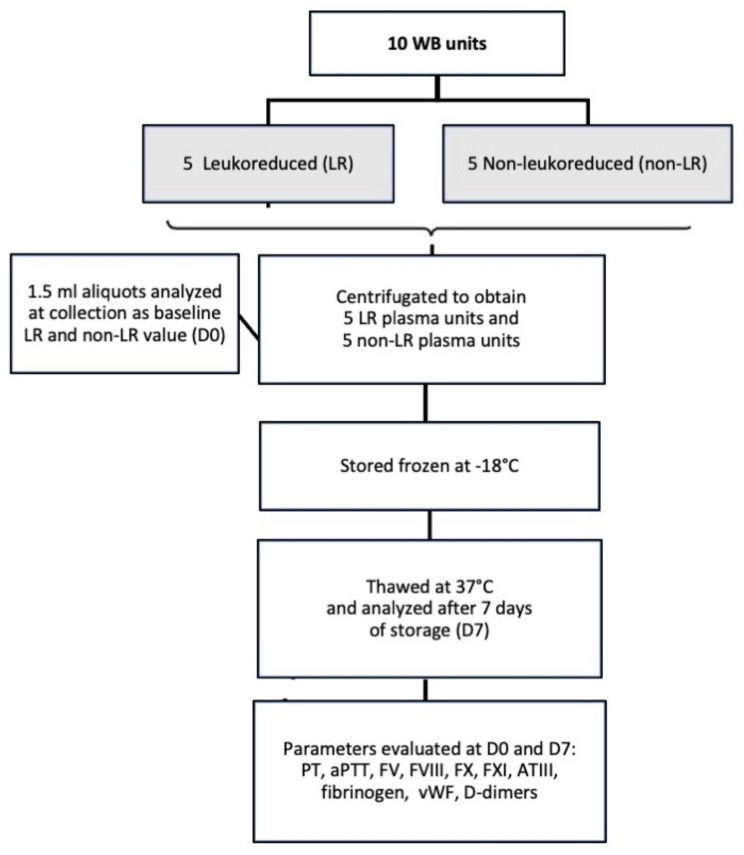
Flowchart summary of the study design in which 5 canine plasma units obtained from leuko-reduced whole blood were evaluated for hemostatic activity and compared to 5 non-leuko-reduced plasma units at time of preparation (baseline value), before storage (D0) and after 7 days of frozen storage (D7).

Within 6 h of blood collection, WB unit centrifugation was performed at 3032× *g* for 20 min at 5 °C in a commercial refrigerated centrifuge designed for blood separation (Rotixa 50RS—Hettich Italia, Milan, Italy) and plasma was expressed from the unit into another attached empty satellite storage bag using a manual plasma extractor. The plasma bags were than separated from the collection system using an electric tube sealer (HemoWeld, Delcon, Arcore, Italy). An aliquot of 1.5 mL of plasma was collected using a sterile connection luer lock needle-free, self-cleaning valve that allowed sterile blood sample collection, and analyzed immediately before storage (D0, baseline value). Each plasma unit was than stored frozen at −18 °C in a dedicated plasma unit freezer (Fiocchetti, Luzzara, RE, Italy), and after 7 days (D7) of frozen storage, was thawed in a warm water bath (37 °C) and a 1.5 mL aliquot was sampled and analyzed. Figure 1 shows a schematic flowchart of the study design.

### 2.2. Laboratory Analysis

All plasma aliquots were placed in polypropylene test tubes and evaluated immediately after sampling. The hemostatic parameters evaluated from each sample collected included: clotting times as prothrombin time (PT) and activated partial thromboplastin time (aPTT), coagulation factor activities for factors V (FV), VIII (FVIII), X (FX), XI (FXI), anticoagulant factor antithrombin III (ATIII), adhesive proteins fibrinogen and von Willebrand factor (vWF) and D-dimer content (DD).

Hemostatic proteins and parameters were measured using the STA Compact Max^®^ analyzer and related commercially available reagents (Stago Italia, Milan, Italy). All tests were performed according to the laboratory standard operating procedures and following the manufacturer’s recommendations.

### 2.3. Statistical Analysis

The data were analyzed using standard descriptive statistics and reported as median ± standard deviation (SD) or median, range and 25th–75th percentiles, depending on their distribution. Normality was established using the Kolmogorov–Smirnov test.

Changes between D0 and D7 in LR and in non-LR units were evaluated using the Wilcoxon test for paired samples. The effect of leukoreduction on hemostatic parameters was evaluated using the Mann–Whitney *U* rank test for comparison of independent nonpaired samples. Differences between groups (D0 and D7, LR and non-LR plasma units) were considered significant at *p* < 0.05, with a confidence interval of 95%.

Statistical analyses were conducted and graphics were created using commercial software (MedCalc^®^ Statistical Software version 20.009, MedCalc Software Ltd., Ostend, Belgium).

## 3. Results

Descriptive analyses (range, median and 25th–75th percentile) relative to each coagulation parameter evaluated at D0 and D7 in LR and non-LR plasma units are reported in Table 1. For technical reasons, D-dimers were not evaluated in one LR plasma unit.

Leuko-reduced plasma units showed a good coagulation factor activity, with median baseline values (D0) ranging from 93% (FVIII) to 80% (FV and vWF). In addition, the median values of most coagulation parameters at D0 were within the canine reference range, with the exception of aPTT and FXI, for which no units had values within the reference range, and for vWF, for which only 80% of units had values within the reference range at D0. All canine non-LR plasma units showed good coagulation factor activity, with median baseline D0 values ranging from 109% (FVIII) to 86% (FV). Median values for most factor activity and coagulation times at D0 were within the canine reference range, with the exception of vWF and DD, for which only 60% of units had values within the reference range at D0.

When changes between D0 and D7 of frozen storage were compared in both LR and non-LR plasma units, median values of PT, aPTT, FV, FVIII, FXI and fibrinogen increased, and FX median values decreased. ATIII median values did not change between D0 and D7 in non-LR, but decreased in LR units. vWF median values decreased in non-LR units, but remained unchanged in LR units. Finally, D-dimers increased in non-LR units and decreased in LR units. However, there were no statistically significant changes during storage between D0 and D7 of frozen storage (Table 2).

**Figure 2:**
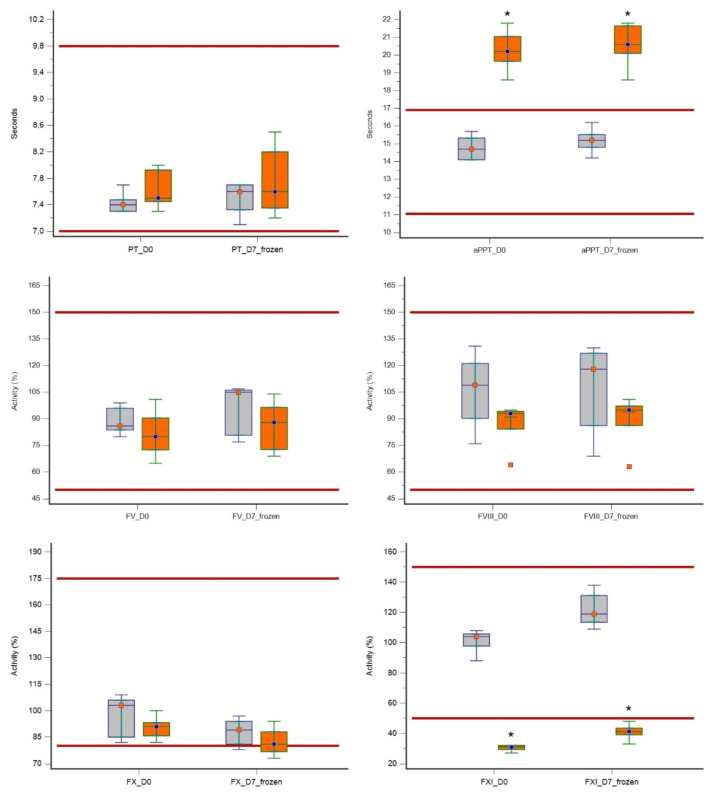

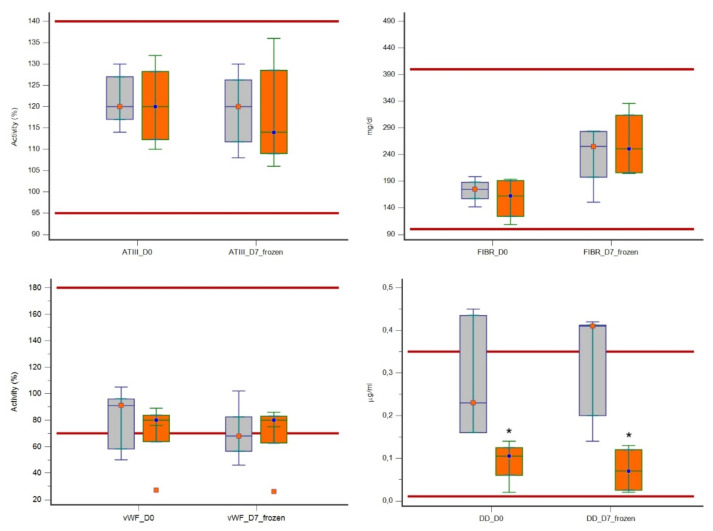
Box-and-whisker plots of hemostatic parameters evaluated in 5 leuko-reduced canine plasma units (orange boxes) compared to 5 non-leuko-reduced plasma units (grey boxes) at baseline (D0) and after 7 days of frozen storage (D7). Asterisks * denote significant differences (*p* < 0.05) in leuko-reduced plasma with respect to non-leuko-reduced plasma units. For each plot, the lower and upper boundaries of the box represent the 25th and 75th percentiles respectively, the horizontal line in the box represents the median and the whiskers represent the range. Outliers are depicted as solitary points. Red lines represent the upper and lower reference ranges.

In evaluating the effect of filtration on coagulation parameters, at both D0 and D7, LR plasma units showed a significantly prolonged aPTT (*p* = 0.0088) and a significantly reduced FXI activity (*p* = 0.0088) as compared with non-LR plasma units. A significantly lower DD value (*p* = 0.0139) was also found in LR units in comparison with non-LR units. There was no statistically significant effect of filtration on the other factors or parameters evaluated (Table 2 and Figure 2). All median values were within the reference range, except for the median values of aPTT and FXI at both D0 and D7 in LR units, which were below the reference range, and values of vWF and DD that were lower and higher respectively, than their reference range at D7.

## 4. Discussion

The processing of whole blood to obtain plasma for transfusion involves a number of steps that can affect the stability of coagulation factors. Filters used in leukoreduction could have a significant impact on the quality of plasma derived from WB filtered through in-line filtration systems. During the filtration process, the contact between coagulation plasma proteins and artificial surfaces could cause changes in the concentration and activity of the coagulation proteins [6,8,9].

In this study, we evaluated the effect of leukoreduction on hemostatic activity of plasma obtained from WB filtered by in-line polyester filters in the specific canine collection system. We showed a significant decrease in FXI and prolongation of aPTT in LR versus non-LR plasma units, both in plasma units before storage and after 7 days of frozen storage. Prolongation of aPTT was presumably due to the low values of FXI. aPTT primarily measures the activity of multiple factors involved in the intrinsic coagulation pathway (factors XII, XI, IX, VIII and fibrinogen), and it has been shown that a significant decrease in any one factor must occur before aPTT becomes significantly prolonged [33]. Differences in the surface coatings of filters could explain the inconsistent adherence of coagulation factors to filters, and the coating of these filter surfaces might contribute to a degree of selectivity in clotting factor adherence. For example, FXI is largely bound to high-molecular-weight kininogen, which has an affinity for negatively charged surfaces, explaining the loss of this protein in some studies [6]. The filter evaluated in our study is a leukocyte reduction filter for single use made from non-woven polyester fibers. However, we do not know whether it is negatively charged. Therefore, we can only speculate that the mechanism by which this filter reduced FXI was the same as demonstrated in human medicine. Reduction of FXI, as well as increased aPTT in leuko-reduced units, has already been demonstrated in some human studies [6,9], but this effect was not found in other studies [7,14,34]. Many published studies in human medicine have investigated the effect of leuko-filtration of either whole blood or plasma on residual clotting factors and shown contradictory results. Evaluation of different leukocyte reduction filters showed that filtration did not influence clotting factor activities in plasma units, and did not activate coagulation [10,11,12,13,14,15,17]. Another human study showed that leukoreduction caused a significant decline in FVIII activity and fibrinogen concentration [8]. We did not observe a consistent influence of filtration on the activity of FVIII in our plasma units. Plasma obtained from our LR WB units did not have a significantly reduced FVIII content at neither D0 nor after 7 days of frozen storage. Indeed, FVIII activity remained above 50% of the initial activity in all plasma samples. This is in agreement with several other human studies that reported good recoveries of factor VIII after filtration [7,9,12,17]. The same was true for fibrinogen content in our study, for which there was no significant difference between LR and non-LR plasma units, as previously shown in one human study [7]. vWF levels were not significantly decreased by leukoreduction in our study, and this is in agreement with previous human studies [7,12,17] which found similar vWF concentrations in the plasma obtained from pre-storage leukocyte-filtrated whole blood. D-dimers are a degradation product of cross-linked fibrin, which forms when FXIII is activated by thrombin [35]. They are markers for reactive fibrinolysis and were not changed by leukocyte filtration [36]. In a human study, plasma filtered through the uncharged filter had moderately reduced levels of D-dimers compared with controls [15], in agreement with the results of our study in which significantly low levels of D-dimers were found in LR plasma units when compared to non-LR units.

In recent decades, leukoreduction has become more common in veterinary transfusion medicine and more studies are being published [20,21,22,24,26,27]. Successful pre-storage leukoreduction of canine blood has also been reported [18,22]. A previous study evaluated the same canine blood collection system and leukoreduction filter used in our study [24]. In that study, the coagulation profiles of PT, aPTT, fibrinogen and coagulation factors VII, VIII, IX, XI and XII, were studied in canine FFP, obtained from LR and non-LR WB pre-cooled units, after 1 year of frozen storage at −30 °C. The only statistically significant difference was a prolonged aPTT in LR compared to non-LR plasma units. However, there was a large variability in FXI and FXII content between units, and no statistical evaluation was reported. It is therefore not possible to know whether, as in our study, the prolonged aPTT in LR units could be due to a reduced FXI content. Another recent study [26] showed no significant differences between LR and non-LR plasma units obtained from non-precooled WB evaluated for aPTT, PT, fibrinogen, antithrombin, protein C and coagulation factors V, VII, VIII, X and XI using a human blood collection and a polyurethane leukocyte filter.

Differences between studies, both in human and veterinary medicine, could potentially be attributed to the type of filter and the length and temperature of storage of WB before leukoreduction [6]. Cardigan et al. [9] examined five different whole-blood filters. Some filters had no effect on any tested coagulation factor activity, including fibrinogen and prothrombin, while others caused a modest increase in median PT or APTT of less than one second. Statistically significant losses in factors V, VIII, IX, XI and XII were observed with 2 of the 5 WB filters. Heiden et al. [14] studied the effect of WB filters from 5 different manufacturers, 4 of which were polyester filters and 1 was a polyurethane filter, and no statistically significant reductions in clotting factors were observed after filtration. Kretzschmar et al. [7], using a polyurethane WB filter, documented a post-filtration increase in aPTT and an associated decline in FVIII:C, but this was only significant after the plasma had been stored at room temperature for 18 h before processing. Therefore, differences in the leukoreduction procedure could also have different effects on coagulation parameters in plasma. Previous veterinary [22] and human studies [7,13] have shown that cooling blood or plasma before leukoreduction improves leukocyte removal and reduces the time needed for leukoreduction (and therefore reduced contact-time of blood with leukocyte filters). In our study, WB was leuko-reduced within 4 h of collection and blood was cooled to 2–6 °C before filtration, as in a previous similar study [24], and differently from that reported in the study by Foote et al. [26] in which WB was not precooled before leukoreduction. Finally, the different results obtained by different veterinary studies may be because the small number of samples analyzed makes it impossible to identify minor differences between groups.

Despite the attention to the study protocol, this study has some limitations. The first one is that there was a small sample size in each of our study groups. Owing to the relatively small sample size, the probability of a type II error is increased, and there might be some significant effects of filtration that we could not detect. However, the number of units analyzed in our study reflects what has previously been reported in veterinary medicine, in which published studies have analyzed numbers of plasma units ranging between 8 and 15 units [18,19,20,21,22,23,24,26,27,28,29,30]. This limited number of units analyzed in veterinary medicine is due to ethical and practical constraints when dealing with a precious resource such as blood and blood components. Furthermore, only one type of whole-blood filter from a single manufacturer was used, whereas different types of leukoreduction filters have different effects on coagulation factors [9,14]. Thus, observations in this study are unique to this manufacturer and the results may not be generalizable to other filter types. Finally, we did not compare the same unit before and after leukoreduction; instead, units from different dogs were compared.

## 5. Conclusions

Our results show that plasma obtained from LR WB has significantly reduced FXI activity and consequently prolonged aPTT. Despite the statistical significance, however, the clinical relevance of low FXI activity in LR plasma units remains to be determined. Further studies with a larger sample size comparing the same unit before and after leukoreduction are needed to determine the clinical significance of these effects.

## Figures and Tables

**Table 1:** Descriptive statistics relating to hemostatic activity in 5 non-leuko-reduced and 5 leuko-reduced canine plasma units evaluated at time of preparation, before storage (baseline value, D0) and after 7 days of frozen storage at −18 °C (D7).

Parameter (Reference Range)	Time	Non-Leuko-Reduced (Non-LR) Plasma				Leuko-Reduced (LR) Plasma			
		Min	Max	Median	25th–75th Percentiles	Min	Max	Median	25th–75th Percentiles
PT (7–9.8 s)	D0	7.3	7.7	7.4	7.3–7.4	7.3	8.0	7.5	7.4–7.9
	D7	7.1	7.7	7.6	7.3–7.7	7.2	8.5	7.6	7.3–8.2
aPPT (11–16.9 s)	D0	14.1	15.7	14.7	14.1–15.3	18.6	**21.8**	**20.2**	19.6–21.0
	D7	14.2	16.2	15.2	14.8–15.5	18.6	**21.8**	**20.6**	20.1–21.6
FV (50–150%)	D0	80.0	99.0	86.0	83.7–96.0	65.0	101.0	80.0	72.5–90.5
	D7	77.0	107.0	105.0	80.7–106.2	69.0	104.0	88.0	72.7–96.5
FVIII (50–150%)	D0	76.0	131.0	109.0	90.2–121.2	64.0	95.0	93.0	84.2–94.2
	D7	69.0	130.0	118.0	86.2–127.0	63.0	101.0	95.0	86.2–97.2
FX (80–175%)	D0	82.0	109.0	103.0	85.0–106.0	82.0	100.0	91.0	85.7–93.2
	D7	78.0	97.0	89.0	81.0–94.0	73.0	94.0	81.0	76.7–88.0
FXI (50–150%)	D0	88.0	108.0	104.0	97.7–105.7	**27.0**	**32.0**	**31.0**	29.2–32.0
	D7	109.0	138.0	119.0	113.5–131.2	**33.0**	**48.0**	**41.0**	39.0–43.5
ATIII (95–140%)	D0	114.0	130.0	120.0	117.0–127.0	110.0	132.0	120.0	112.2–128.2
	D7	108.0	130.0	120.0	111.7–126.2	106.0	136.0	114.0	109.0–128.5
Fibrinogen (100–400 mg/mL)	D0	141.8	198.6	174.6	157.3–188.0	108.5	193.7	162.3	123.9–191.2
	D7	150.7	284.2	255.3	197.5–283.4	204.6	336.1	250.8	205.8–313.7
vWF (70–180%)	D0	50.0	105.0	91.0	58.2–96.0	**27.0**	89.0	80.0	63.7–83.7
	D7	46.0	102.0	**68.0**	56.5–82.5	**26.0**	86.0	80.0	62.7–83.0
D-dimers (0.01–0.35 μg/mL)	D0	0.16	**0.45**	0.23	0.16–0.43	0.02	0.14	0.10	0.06–0.12
	D7	0.14	**0.42**	**0.41**	0.20–0.41	0.02	0.13	0.07	0.02–0.12

Min: minimum, max: maximum; PT: prothrombin time; aPTT: activated partial thromboplastin time; FV: factor V; FVIII factor VIII; FX: factor X; FXI: factor XI; ATIII: antithrombin III; vWF: von Willebrand factor. Numbers in bold are values outside the canine normal reference range.

**Table 2:** Comparison of coagulation parameters in 5 canine non-leuko-reduced and 5 leuko-reduced plasma units evaluated at baseline (D0) and after 7 days of frozen storage at −18 °C (D7).

Variable (Reference Range)	Time Point	Non-Leuko-Reduced (Non-LR) Plasma Units				Leuko-Reduced (LR) Plasma Units				*p-*Value for Comparison Non-LR and LR Plasma Units
		Median	Average Rank	Paired Difference D0 vs. D7		Median	Average Rank	Paired Difference D0 vs. D7		
				Median	*p-*Value			Median	*p-*Value	
PT (7–9.8 s)	D0	7.4	4.2	0.0	-	7.5	6.8	0.10	0.4375	0.1666
	D7	7.6	5.0			7.6	6.0			0.5982
aPTT (11–16.9 s)	D0	14.7	3.0	0.5	0.1250	20.2	8.0	0.4	-	0.0088
	D7	15.2	3.0			20.6	8.0			0.0088
FV (50–150%)	D0	86.0	6.3	8.0	0.3125	80.0	4.7	4.0	0.3125	0.4020
	D7	105.0	6.8			88.0	4.2			0.1745
FVIII (50–150%)	D0	109.0	7.1	−1.0	0.8125	93.0	3.9	1.0	0.1875	0.0937
	D7	118.0	6.4			95.0	4.6			0.3472
FX (80–175%)	D0	103.0	6.3	−8.0	0.0625	91.0	4.7	−9.0	0.0625	0.4005
	D7	89.0	6.5			81.0	4.5			0.2948
FXI (50–150%)	D0	104.0	8.0	21.0	0.0625	31.0	3.0	9.0	0.0625	0.0088
	D7	119.0	8.0			41.0	3.0			0.0088
ATIII (95–140%)	D0	120.0	5.7	−1.0	-	120.0	5.3	−3.0	0.3125	0.8340
	D7	120.0	5.6			114.0	5.4			0.9168
Fibrinogen (100–400 mg/dl)	D0	174.6	6.2	80.7	0.125	162.3	4.8	97.8	0.0625	0.4647
	D7	255.3	5.2			250.8	5.8			0.7540
vWF (70–180%)	D0	91.0	6.4	−4.0	0.0625	80.0	4.6	−1.0	-	0.3472
	D7	68.0	5.0			80.0	6.0			0.6015
D-Dimers (0.01–0.35 μg/mL)	D0	0.23	7.00	−0.01	0.8125	0.10	2.50	−0.01	-	0.0139
	D7	0.41	7.00			0.07	2.50			0.0139

PT, prothrombin time; aPTT, activated partial thromboplastin time; FV, factor V; FVIII, factor VIII; FX, factor X; FXI, factor XI; ATIII, antithrombin III; FIBR, fibrinogen; DD, D-dimers; vWF, von Willebrand factor. *p*-values in bold are statistically significant at *p* < 0.05.

## Data Availability

The datasets generated for this study are available upon request to the corresponding author.
